# Adhesion and friction of the smooth attachment system of the cockroach *Gromphadorhina portentosa* and the influence of the application of fluid adhesives

**DOI:** 10.1242/bio.024620

**Published:** 2017-05-15

**Authors:** Oliver Betz, Melina Frenzel, Michael Steiner, Martin Vogt, Malte Kleemeier, Andreas Hartwig, Benjamin Sampalla, Frank Rupp, Moritz Boley, Christian Schmitt

**Affiliations:** 1 Institut für Evolution und Ökologie, Universität Tübingen, Auf der Morgenstelle 28, Tübingen D-72076, Germany; 2 Fraunhofer-Institut für Fertigungstechnik und Angewandte Materialforschung, Wiener Str. 12, Bremen D-28359, Germany; 3 Universität Bremen, Fachbereich 2 Biologie/Chemie, Leobener Str., Bremen 28359, Germany; 4 University Hospital Tübingen, Section Medical Materials Science and Technology, Osianderstr. 2-8, Tübingen D-72076, Germany

**Keywords:** Adhesion, Adhesion-mediating secretion, Anisotropy, Insect, Locomotion, Tribometry

## Abstract

Two different measurement techniques were applied to study the attachment of the smooth foot pads of the Madagascar hissing cockroach *Gromphadorhina portentosa*. The attachment of the non-manipulated adhesive organs was compared with that of manipulated ones (depletion or substitution by artificial secretions). From measurements of the friction on a centrifuge, it can be concluded that on nanorough surfaces, the insect appears to benefit from employing emulsions instead of pure oils to avoid excessive friction. Measurements performed with a nanotribometer on single attachment organs showed that, in the non-manipulated euplantulae, friction was clearly increased in the push direction, whereas the arolium of the fore tarsus showed higher friction in the pull direction. The surface of the euplantulae shows an imbricate appearance, whereupon the ledges face distally, which might contribute to the observed frictional anisotropy in the push direction. Upon depletion of the tarsal adhesion-mediating secretion or its replacement by oily fluids, in several cases, the anisotropic effect of the euplantula disappeared due to the decrease of friction forces in push-direction. In the euplantulae, adhesion was one to two orders of magnitude lower than friction. Whereas the tenacity was slightly decreased with depleted secretion, it was considerably increased after artificial application of oily liquids. In terms of adhesion, it is concluded that the semi-solid consistence of the natural adhesion-mediating secretion facilitates the detachment of the tarsus during locomotion. In terms of friction, on smooth to nanorough surfaces, the insects appear to benefit from employing emulsions instead of pure oils to avoid excessive friction forces, whereas on rougher surfaces the tarsal fluid rather functions in improving surface contact by keeping the cuticle compliable and compensating surface asperities of the substratum.

## Abstract (precis)

**Summary:** Friction and adhesion of a cockroach are analysed in an experimental approach that focuses on the function of the tarsal adhesion-mediating secretion.

## INTRODUCTION

In the past few years, cockroaches (Blattodea) have become an accepted study object in the field of bioadhesion (e.g. Clemente and Federle, 2008; Clemente et al., 2009; Lepore et al., 2013; Gerhardt et al., 2015, 2016; Betz et al., 2016), they possess large smooth adhesive pads and have shown good climbing performance in previous studies (e.g. Roth and Willis, 1952; Goldman et al., 2006; Clemente and Federle, 2008). In the current study, we investigated the large and heavy Madagascar hissing cockroach (∼5-10 g, leading to a normal force of 8-16 mN per tarsus) *Gromphadorhina portentosa* (Schaum, 1853) to further contribute to our understanding of the locomotory and attachment performance of cockroaches. This species was selected, because its tarsal adhesion-mediating secretion has recently been analysed and functionally interpreted. Gerhardt et al. (2015) have analysed the adhesion-mediating secretion produced by the tarsal euplantulae of this species and established the presence of n-alkanes and methyl-branched alkanes in the range of C27-C33. The long chain lengths >C20 combined with branched compounds suggest a semi-solid, grease-like consistency of the adhesion-mediating secretion at ambient temperature and humidity conditions experienced by the animals. Comparisons between tarsi and tibiae have revealed an increase of n-C29 and 3-Me-C29 in the tarsal adhesion-mediating secretion, whereas all triacontanes (both methyl-branched and the *n*-alkane) are decreased relative to tibia cuticular samples (Gerhardt et al., 2016). In addition, by combining Fourier transform infrared spectroscopy, sodium dodecyl sulphate polyacrylamide gel electrophoresis and matrix-assisted laser desorption/ionization mass spectrometry, Betz et al. (2016) have detected several peptides and proteins (some of them probably bound to saccharides) and discussed their possible functions in the secretion. Another reason for selecting *G. portentosa* in our study was the recently established structural evidence for the function of its attachment pads in producing, storing, and secreting an adhesion-mediating secretion and releasing it to the exterior (C. S. and O. B., unpublished data).

In the smooth adhesion pads of insects, both adhesion and friction are known to be influenced by the tarsal adhesion-mediating secretion that works in concert with the viscoelasticity of the cuticular material (e.g. Gorb et al., 2000; Drechsler and Federle, 2006; Perez Goodwyn et al., 2006; Gorb, 2007; Clemente and Federle, 2008; Scholz et al., 2008; Betz, 2010; Dirks et al., 2010; Bußhardt et al., 2012). The aim of the present study has been to quantify the attachment ability of the non-manipulated smooth adhesive tarsal pads of the Madagascar hissing cockroach (1) having all tarsi attached to the ground, (2) of the arolium of the fore and hind leg and of the euplantula of the fore, middle and hind leg, and (3) considering possible direction-dependent anisotropies in terms of friction. Moreover, in order to understand the interaction between the morphology of the smooth attachment system and the adhesion-mediating secretion, the attachment of the non-manipulated tarsi has been compared with setups in which the tarsal adhesion-mediating secretion has been experimentally depleted or subsequently replaced by the hydrocarbon squalane or a synthetic water-in-oil (w/o) emulsion that we had previously prepared from squalane and gelatin in the framework of a comparative study on the adhesion and friction of bioinspired synthetic ‘insect adhesives’ (Speidel et al., 2017).

## RESULTS

### Surface roughness and surface free energy of test substrates

The roughness parameters of the three types of Al_2_O_3_ grinding discs amounted to 0.05 µm (‘nanorough’), 3 µm (‘microrough’), and 11 µm (‘rough’) (Table 1). The apparent surface energies and polarities of the test surfaces, as calculated from the contact angle measurements, are summarized in Table 2. They show that all three surfaces are better wettable with non-polar substances (such as diiodomethane) compared with polar ones. However, in contrast to both the other surfaces, the 11-µm surface shows a higher surface polarity and an improved wettability towards polar liquids such as water.

**Table 1.**
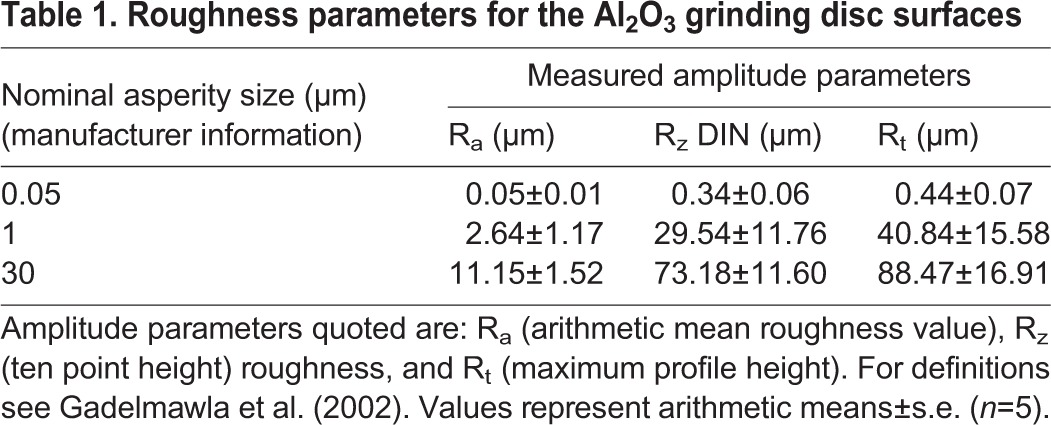
**Roughness parameters for the Al_2_O_3_ grinding disc surfaces**

**Table 2.**
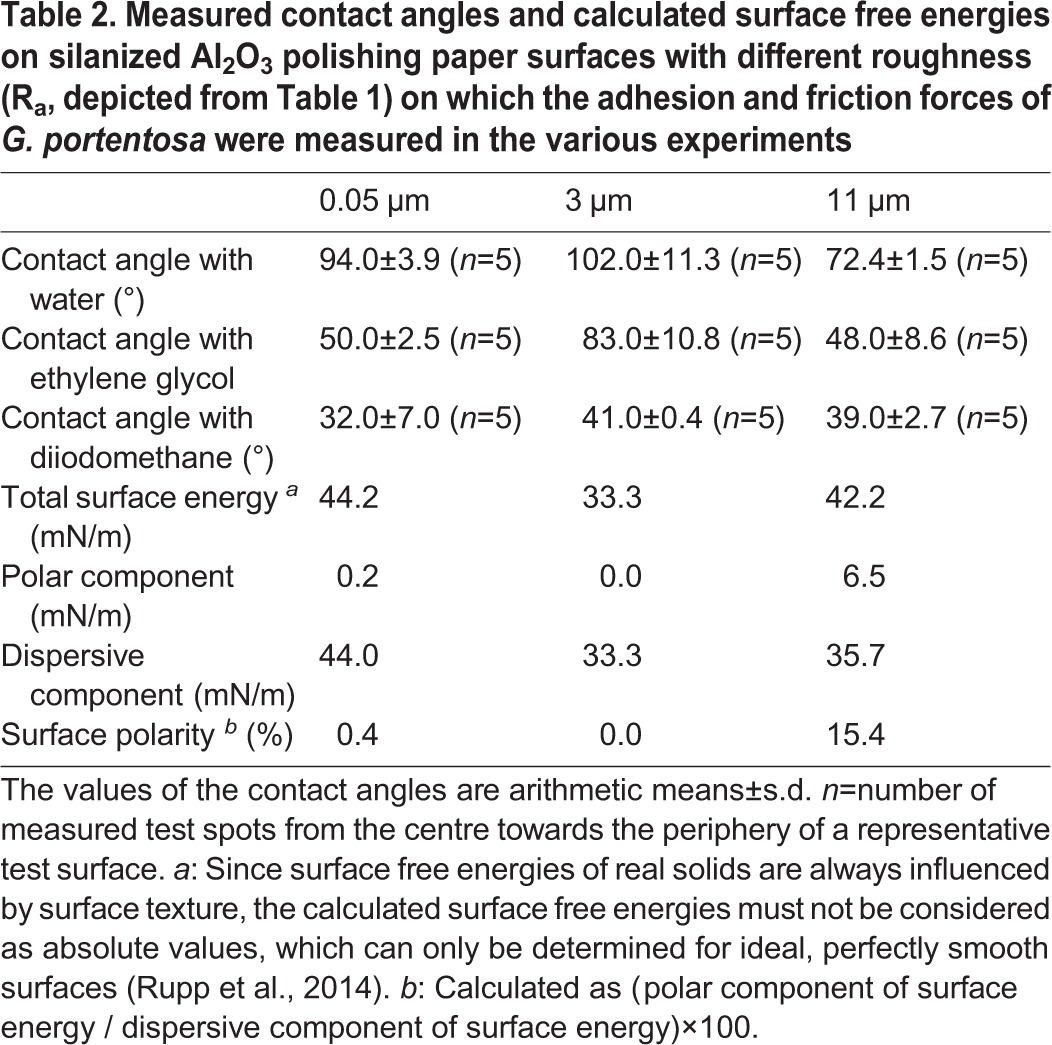
**Measured contact angles and calculated surface free energies on silanized Al_2_O_3_ polishing paper surfaces with different roughness (R_a_, depicted from Table 1) on which the adhesion and friction forces of *G. portentosa* were measured in the various experiments**

### External tarsal morphology of *G. portentosa*

**Fig. 1.**
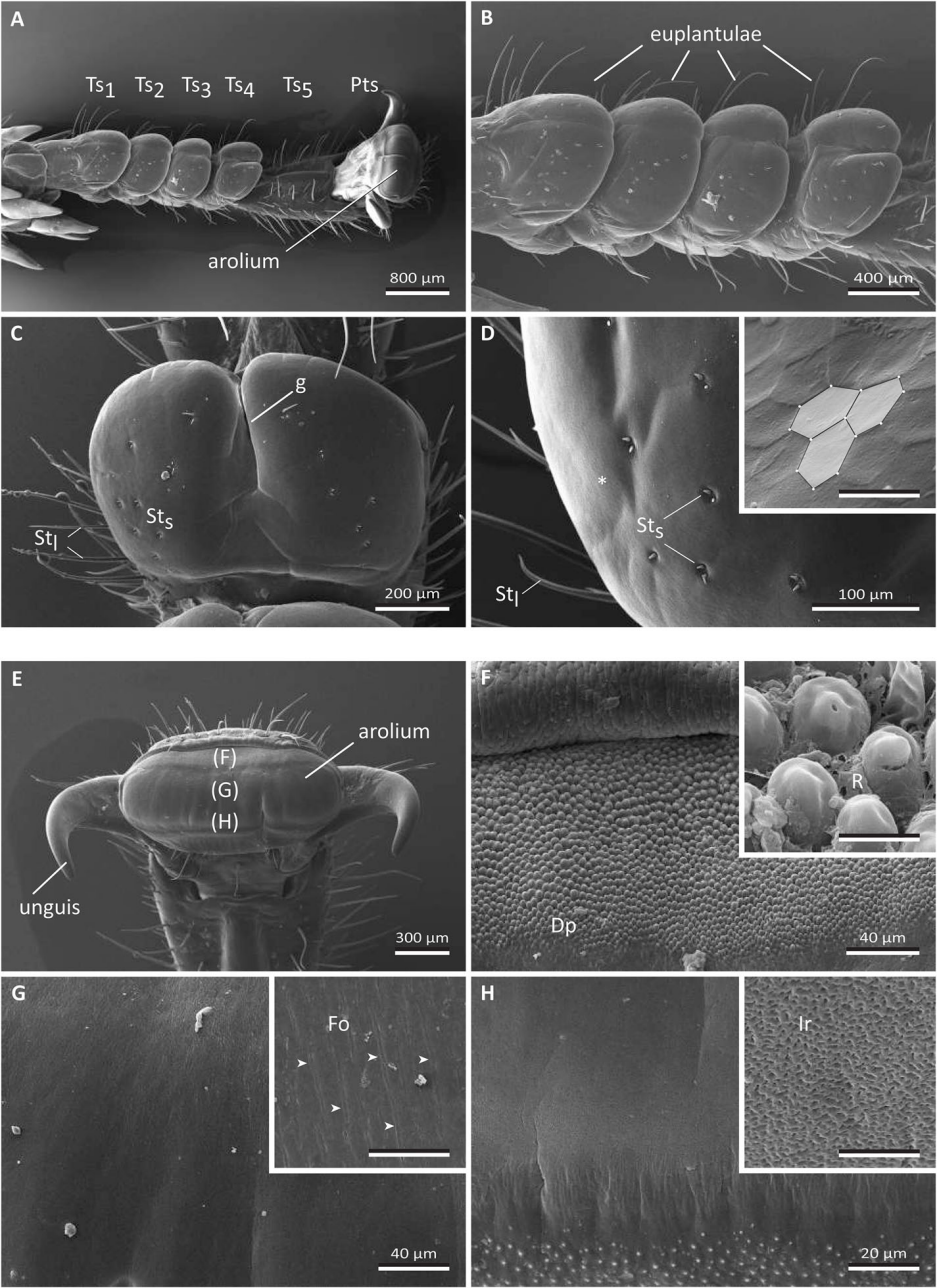
**Scanning electron microscopy images of the ventral tarsus of the Madagascar hissing cockroach *Gomphadorhina portentosa.*** (A-D) Fore tarsus. (E-H) Middle tarsus. The inset in D is taken from a tarsus that was additionally cleaned by hydrogen peroxide, so that its hexagonal surface pattern became visible. In A the general overview of a cockroach tarsus is shown. It consists of five tarsomeres, of which the first four bear a euplantulum (A-D), and a terminal pretarsus with an arolium in between the rigid ungues (E-H). The inset in D is taken from a tarsus that was additionally cleaned with hydrogen peroxide to reveal its hexagonal pattern. All the other images were prepared without additional cleaning. The capital letters in parenthesis within E indicate the area of the arolium surface depicted in the following figure parts F-H. The insets in F-H give a detailed view in higher magnification that is representative for the corresponding part of the arolium displayed in E. Dp, digitiform projections; Fo, folds; g, central groove dividing the euplantulum into two distinct lobes; Ir, cuticular irregularities; Pts, pretarsus; R, residues of the tarsal adhesion-mediating secretion; St_l_, sensilla trichodea long type; St_s_, sensilla trichodea short type; Ts_1-5_, tarsomeres 1-5. White arrows in G indicate position of the folds. Scale bars of the detailed views: 5 µm.

The tarsi of *G. portentosa* consist of five tarsomeres (Ts_1_-Ts_5_) and a distal pretarsus (Pts) (Fig. 1A). The ventral side of each subsegment (except for the fifth tarsomere) possesses a smooth and highly compressible adhesive pad (Fig. 1A-E). The four pads associated with the first four tarsomeres are called euplantulae (Fig. 1A-D), whereas the terminal trapeziform pad associated with the pretarsus is termed an arolium (Fig. 1A,E-H).

The euplantulae are divided by a central longitudinal groove (g) into a right and a left lobe. The depth of this groove continuously increases from proximal to distal and is most pronounced in the fourth tarsomere that therefore consists of a pair of pads rather than one single pad (Fig. 1B,C). Long sensory setae (sensilla trichodea, long type) are located on the lateral sides of the euplantulae and arise from sclerotized cuticular parts in the transition area to the soft cushions (St_l_: Fig. 1A-D). The surface of the euplantulae is mostly smooth and unstructured, apart from small concavities situated laterally from the longitudinal groove, where the tips of the paired mechanosensilla (sensilla trichodea, short type) are located (St_s_:Fig. 1C-D). Close to the lateral margin of the euplantulae, the cuticle surface has an imbricate appearance (Fig. 1D). A hexagonal pattern covered the ventral euplantulae (inset in Fig. 1D), and differed in both prominence and site of cuticular patterning among the analysed individuals. The ledges of these structures faced medio-distally.

The pretarsus bears the terminal arolium, which is flanked by a pair of claws (ungues) (Fig. 1E). The surface of this adhesive pad can be sub-divided into three distinct areas. On its most distal margin, the surface is characterized by a transverse band of digitiform projections (Dp: Fig. 1F). Residues of the tarsal adhesion-mediating secretion can be found between them (R: Fig. 1F). Further proximally, narrow, longitudinally oriented fissures follow (Fig. 1E,G). At its proximal end, the arolium is structured by dense irregularities in the submicron range (Fig. 1E,H).

### Influence of test surface roughness and tarsal adhesion-mediating secretion on cockroach attachment

**Fig. 2.**
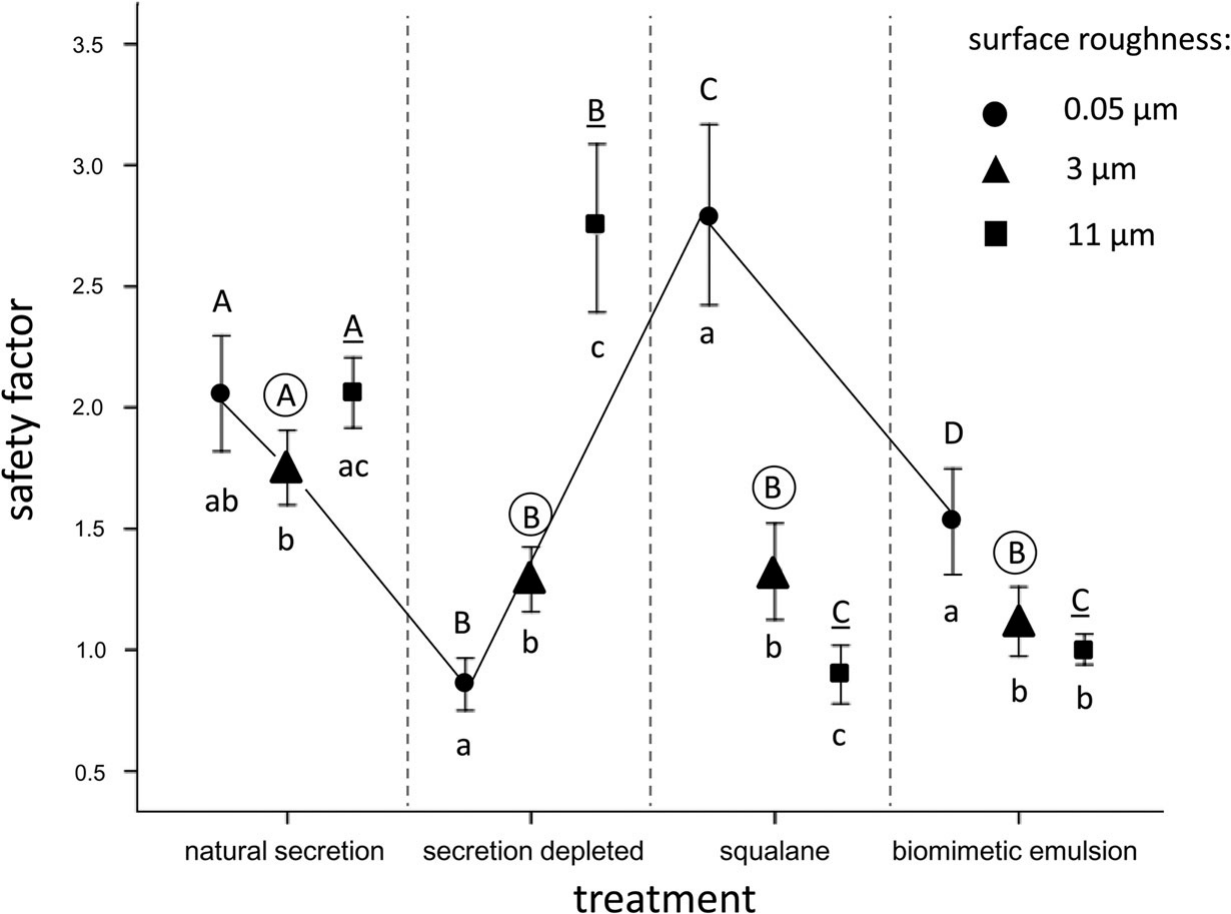
**Safety factors obtained in friction measurements with a centrifugal force device for four tarsal treatments on silanized Al_2_O_3_ grinding disc surfaces with three different roughnesses.** Arithmetic means and standard errors are shown. For the definition of the safety factor, see Eqn 1 in the text. Upper case letters above the bars are indicative of significant differences between the different treatments at identical surface roughnesses (*P*<0.05, paired *t*-tests, *n*=20). Upper case letters of the same format (i.e. regular, encircled, underlined) should be compared between the four different treatments, since they respectively represent identical surface roughnesses (as exemplified by the black line connecting the values obtained on 0.05-µm-rough surfaces for different tarsal treatments). Different lower case letters below the bars are indicative of significant differences between the different roughnesses within a certain treatment (*P*<0.05, paired *t*-tests, *n*=20).

The linear mixed model for repeated measures revealed a significant influence of the two-way interaction term between treatment and roughness (Table 3). The results of this experiment are summarized in Fig. 2. The means of the safety factors obtained from the centrifugal force experiments varied from 0.9 (no secretion combined with the nanorough surface) to 2.8 (squalane combined with the nanorough surface). In non-manipulated tarsi that retained their natural secretion, the mean safety factors lay between 1.8 and 2.1. In this case, friction was only slightly reduced at the middle roughness of 3 µm nominal asperity size (Fig. 2). The depletion of the tarsal secretion considerably decreased friction on the nanorough and the middle-rough surface, whereas it largely increased friction on the rough surface. Application of the fluid adhesives, i.e. squalane and the bioinspired emulsion, significantly influenced friction in comparison with the natural secretion. Interestingly, on the nanorough surface, application of the adhesives led to increased friction values, whereby squalane attained much higher friction values (even higher than the natural adhesive) than the bioinspired emulsion.

**Table 3.**
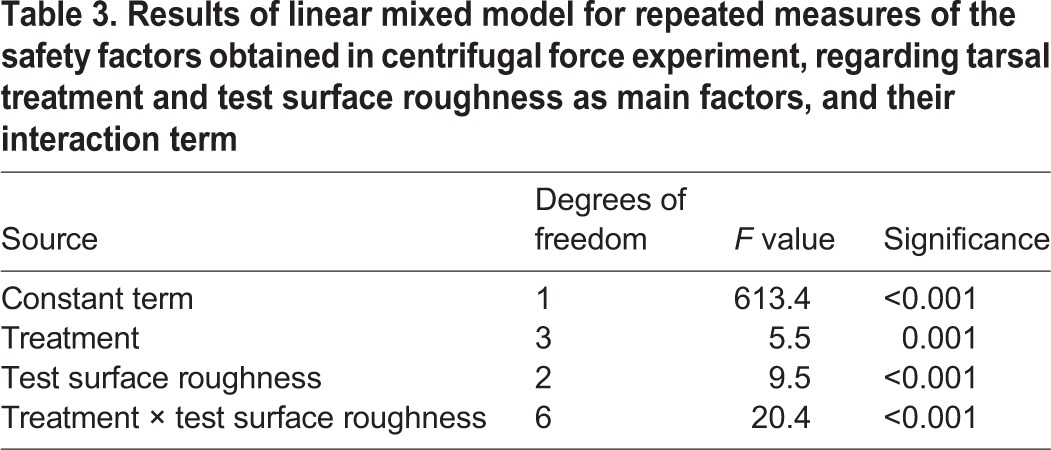
**Results of linear mixed model for repeated measures of the safety factors obtained in centrifugal force experiment, regarding tarsal treatment and test surface roughness as main factors, and their interaction term**

### Influence of tarsal adhesion-mediating secretion on the adhesion and friction of a single euplantulum

#### Adhesion

**Fig. 3.**
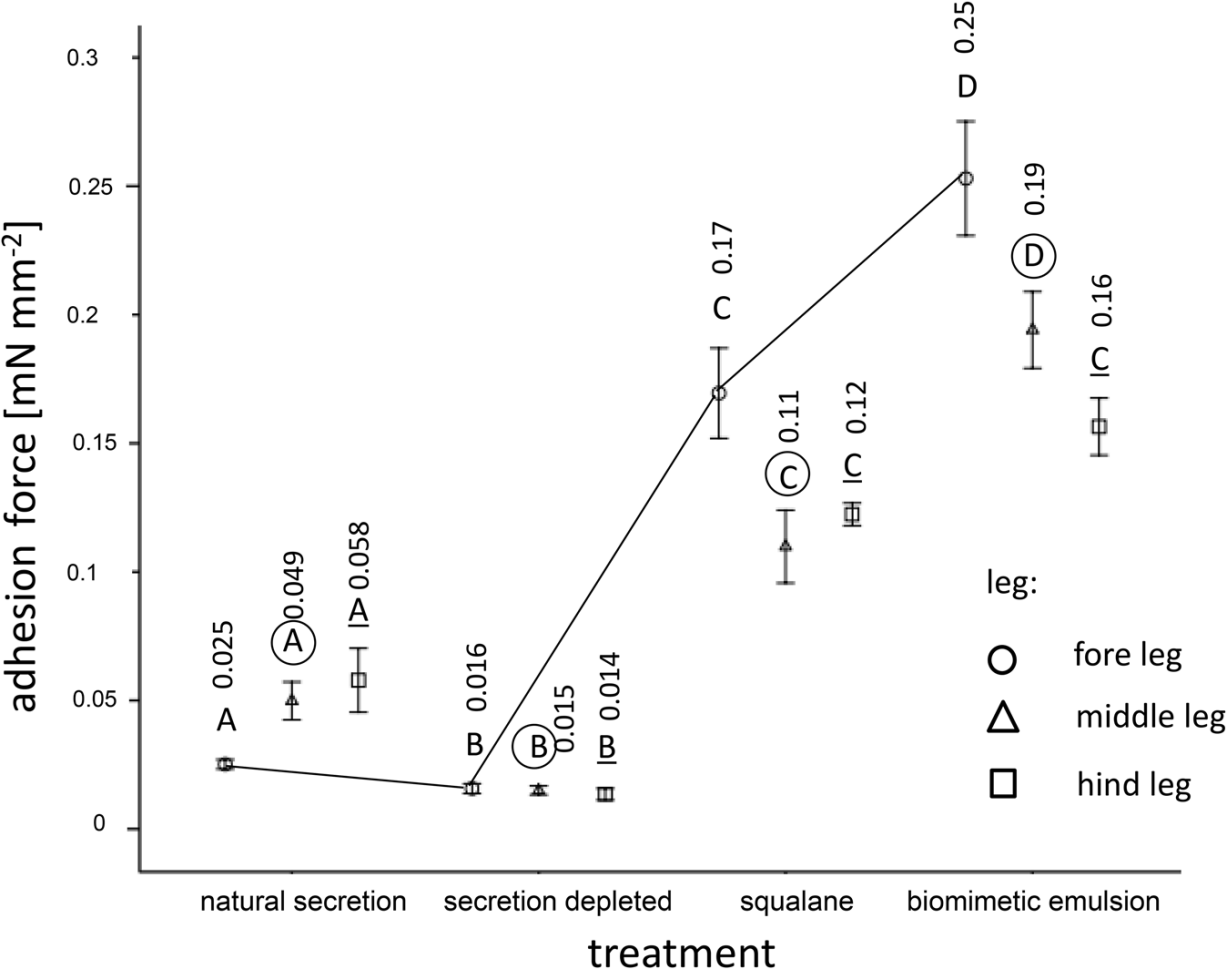
**Adhesion measured with the nanotribometer on euplantulae on silanized Al_2_O_3_ grinding disc surfaces showing a roughness of 3 µm.** Arithmetic means (their exact numbers are given above each bar) and standard errors are shown. Different upper case letters above the bars are indicative of significant differences between the different treatments at identical segmental positions of legs (i.e. for, middle, hind) (*P*<0.05, paired Wilcoxon tests, *n*=10). Only upper case letters of the same format (i.e. regular, encircled, underlined) should be compared between the four different treatments, since they respectively represent identical legs (as exemplified by the black line connecting the segmental fore leg positions of the different treatments). Different segmental position of legs (i.e. fore, middle, hind) are indicated by different symbols at the bars. The given reference surface area (1 mm²) refers to the estimated contact surface area with the tester and not the real one.

For the applied normal load of 8 mN, our calibration curves revealed the following pooled contact areas of the euplantulae (mm²) (♂/♀): fore tarsus: 2.71/2.14; middle tarsus: 4.25/3.17; hind tarsus: 5.38/4.03. For the following calculations, we related the measured adhesive forces to these contact areas. The linear mixed model for repeated measures revealed the significant influence of the two-way interaction between treatment and the segmental position of leg (i.e. fore, middle, hind) (Table 4). The results of this experiment are summarized in Fig. 3. The means of the adhesion forces varied from 13.5 µN mm^−2^ (no secretion combined with the hind tarsus) to 253.0 µN mm^−2^ (bioinspired emulsion combined with the fore tarsus). In non-manipulated tarsi that retained their natural secretion, the mean adhesion force lay between 25.1 and 57.9 µN mm^−2^. Whereas the depletion of the tarsal adhesion-mediating secretion significantly reduced the adhesive strength by the two- to fourfold with respect to the natural adhesive, the replacement of the natural adhesive by squalane and the synthetic emulsion significantly increased adhesion. Here, the synthetic emulsion containing squalane gave higher adhesion forces than pure squalane.

**Table 4.**
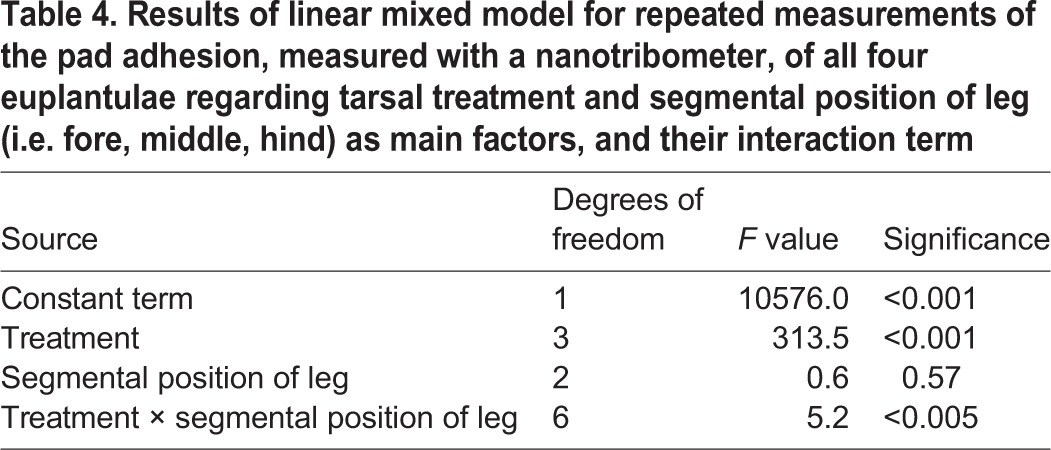
**Results of linear mixed model for repeated measurements of the pad adhesion, measured with a nanotribometer, of all four euplantulae regarding tarsal treatment and segmental position of leg (i.e. fore, middle, hind) as main factors, and their interaction term**

#### Friction

**Fig. 4.**
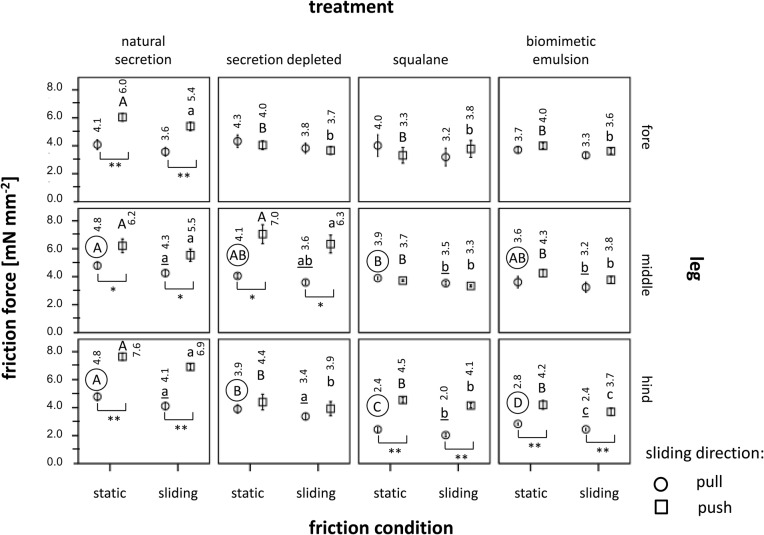
**Friction measured with the nanotribometer on the euplantulae on Al_2_O_3_ grinding disc surfaces showing a roughness of 3 µm.** Arithmetic means (their exact numbers are given above each bar) and standard errors are shown. Different letters above the bars are indicative of significant differences between the different treatments at identical sliding regimes (*P*<0.05, paired Wilcoxon tests, *n*=10). Uppercase letters are used for static friction, lowercase letters for sliding friction. Only letters of the same format (upper and lower case, regular, encircled) within the same leg (i.e. fore, middle, hind), sliding regime (i.e. static or sliding) and sliding direction (i.e. push or pull) should be compared between the four different treatments. Different sliding directions are indicated by different symbols at the bars. The asterisks below two bars, respectively, are indicative of significant differences between the pull (circle) and the push (square) direction. *P<0.05, **P<0.01. The given reference surface area (1 mm²) refers to the estimated contact surface area with the tester and not the real one.

The linear mixed model for repeated measures revealed a significant influence of the three-way interaction term between treatment, segmental position of leg (i.e. fore, middle, hind), and sliding direction (Table 5). In the following, we conducted pairwise comparisons between both the sliding directions (push versus pull) and the four treatments, keeping all the other factors (e.g. sliding regime, segmental position of leg) constant (Fig. 4). The non-manipulated fore, middle and hind tarsi that retained their natural adhesive showed a clear frictional anisotropy, with 1.3-1.7 times higher friction forces in the distal (push) direction compared with the proximal (pull) direction. Whereas in the manipulated tarsi, most cases exhibited no difference between both the established sliding directions, the same anisotropic behaviour was observed as in the non-manipulated tarsi in three cases (Fig. 4).

**Table 5.**
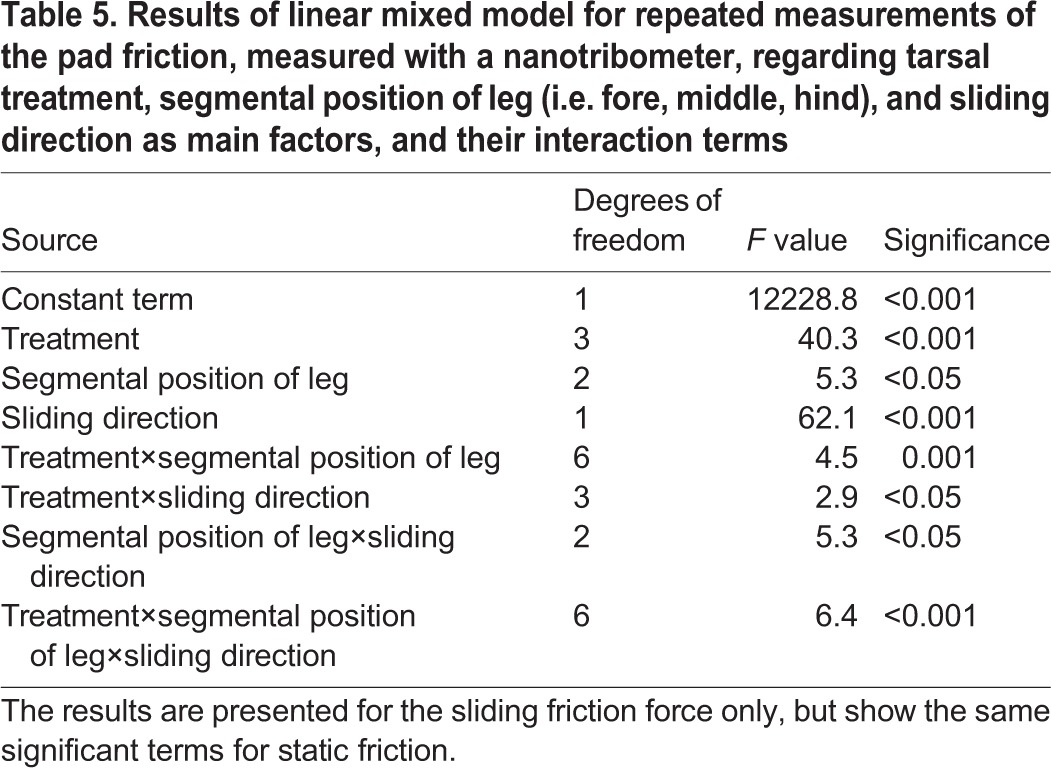
**Results of linear mixed model for repeated measurements of the pad friction, measured with a nanotribometer, regarding tarsal treatment, segmental position of leg (i.e. fore, middle, hind), and sliding direction as main factors, and their interaction terms**

Because of an overall correspondence between the pattern of the static and the sliding friction regimes, we consider only sliding friction in the following. In the non-manipulated tarsi that retained their natural secretion, the mean sliding friction force laid between 3.6-4.3 mN mm^−2^ (pull direction) and 5.4-6.9 mN mm^−2^ (push direction). In the fore and hind tarsi, the depletion of the adhesion-mediating secretion reduced the friction force by about twofold with respect to the natural adhesive. However, this was only the case for the push direction, whereas in the pull direction, such reduction could not be established.

On application of the fluid adhesives, i.e. squalane and the bioinspired emulsion, friction was significantly impaired in comparison with the natural secretion (1.2-2.0 times in the pull direction and 1.3-1.9 times in the push direction); it showed an overall resemblance to the reduced friction of the fore and hind tarsi whose adhesion-mediating secretion had been depleted (Fig. 4).

### Comparison of friction anisotropy between euplantula and arolium

**Fig. 5.**
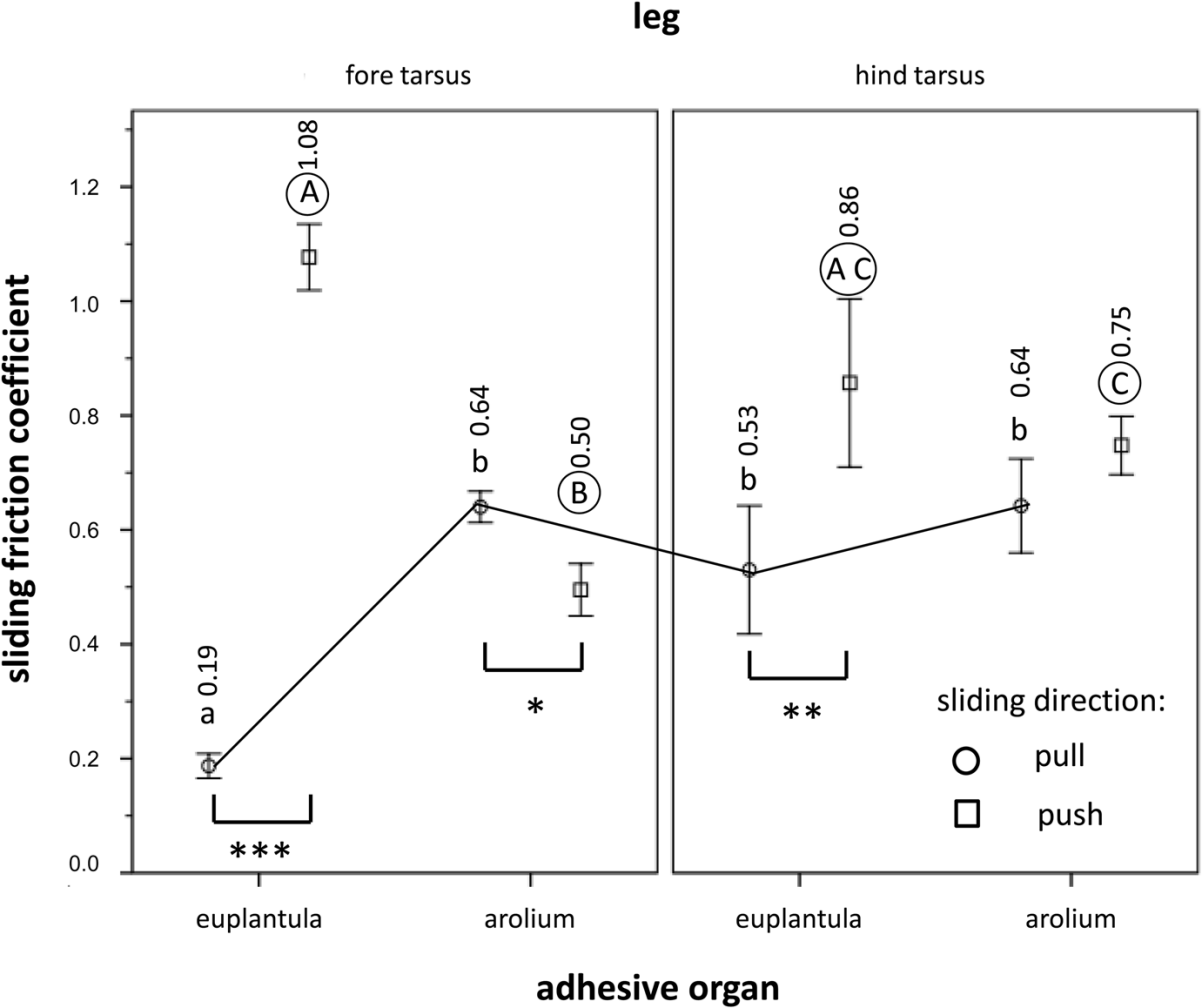
**Friction measured with the nanotribometer with respect to the comparison between euplantula and arolium in the fore and the hind tarsus on the Al_2_O_3_ grinding disc surfaces showing a roughness of 3 µm.** Arithmetic means (their exact numbers are given above each bar) and standard errors are shown. Different letters above the bars are indicative of significant differences between the adhesive organs and their segmental position of leg (i.e. fore, middle, hind) at identical sliding regimes [*P*<0.05, paired Wilcoxon tests, *n*=14 (in the case of the arolium of the hind tarsus) and *n*=10 (in all other cases)]. In the case of the arolium of the hind tarsus, the four additional individuals were used to test for possible differences between push and pull of this arolium. Lowercase letters are used for the pull direction, encircled uppercase letters for the push direction. Only letters of the same format within the same sliding direction (i.e. push or pull) should be compared (as exemplified by the black line connecting the different adhesive organs in pull direction). Different sliding directions are indicated by different symbols at the bars. The asterisks below two bars, respectively, are indicative of significant differences between the pull (circle) and the push (square) direction. *P<0.05, **P<0.01, and ***P<0.005.

The linear mixed model for repeated measures revealed a significant influence of the three-way interaction term between segmental position of leg (i.e. fore, middle, hind), adhesive organ, and sliding direction (Table 6). Thus, in the following, we conducted paired comparisons between the sliding directions (push versus pull) and the adhesive organs (euplantula versus arolium), keeping the other variables constant (Fig. 5). Except for the arolium of the hind tarsi, the adhesive organs showed a clear frictional anisotropy. In the case of the euplantula, this amounted to a factor of 5.7 (fore tarsus) to 1.6 (hind tarsus) in the distal (push) direction compared with the proximal (pull) direction. In contrast, in the arolium of the fore tarsus, the friction was 1.3 times higher in the pull compared with the push direction. In the pull direction, the arolium of the fore tarsus showed a much higher friction than the euplantula of this tarsus. Vice versa, in the push direction, the euplantula of the fore tarsus exhibited a significantly higher friction coefficient than the arolium. The effects regarding the anisotropic behaviour of the arolium and the differences between the two adhesive organs could not be observed in the hind tarsus.

**Table 6.**
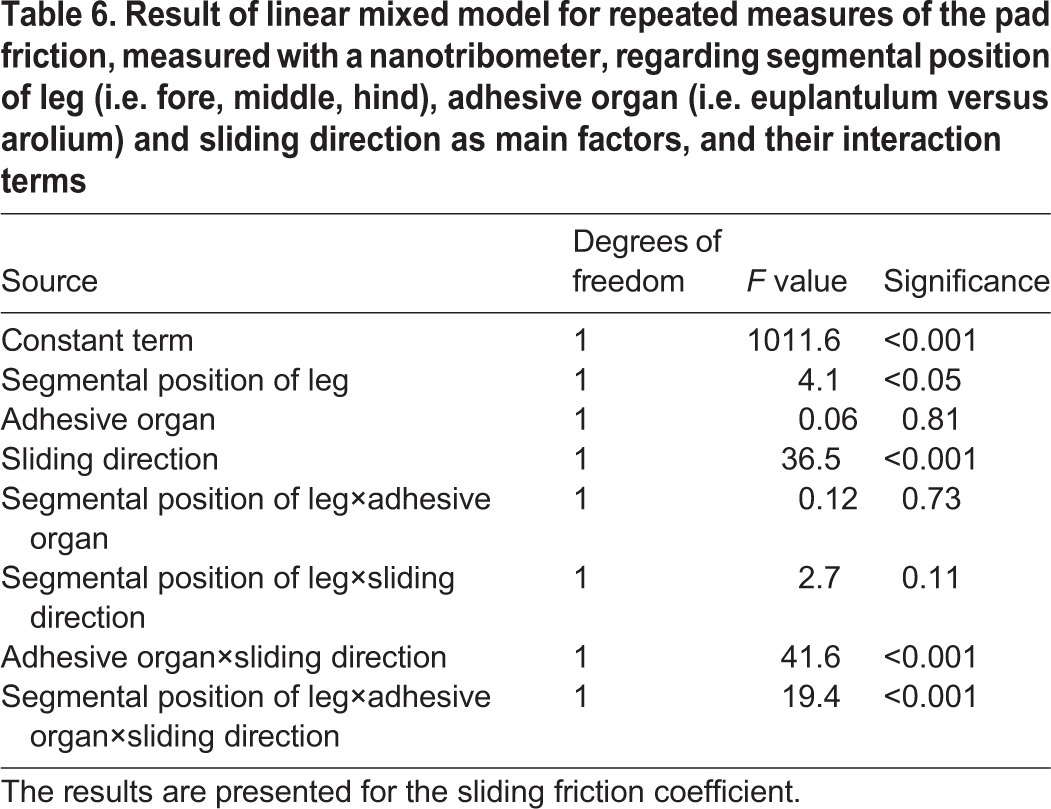
**Result of linear mixed model for repeated measures of the pad friction, measured with a nanotribometer, regarding segmental position of leg (i.e. fore, middle, hind), adhesive organ (i.e. euplantulum versus arolium) and sliding direction as main factors, and their interaction terms**

## DISCUSSION

The experiments presented in this study encompass aspects such as friction, adhesion, surface roughness and the influence of the tarsal adhesion-mediating secretion and its interaction with the compliant viscoelastic pad cuticle of the euplantulae of the cockroach *G. portentosa*. By comparing the friction of non-manipulated euplantulae (that have retained their natural secretion) with euplantulae whose adhesion-mediating secretion had been depleted or replaced by synthetic adhesive fluids, we expected to gain further insights into the functional role of the tarsal adhesion-mediating secretion in insects. Our analyses do not include the effects of the tarsal chain with its natural mobility, which can also have an effect on force directionality (e.g. Clemente et al., 2009; Bußhardt et al., 2012). Although the film thickness of the adhesion-mediating secretion is not directly measurable, the ‘film thickness’ applied in our experiments should be comparable to that used by insects for the following reasons: (1) For the centrifuge experiments, we let the tarsi of the cockroaches only shortly touch a fluid-impregnated filter paper, so that the applied film was very thin. Cryo-SEM control images did not reveal any visible difference between the this way manipulated tarsal pads and the non-manipulated ones, whose adhesion-mediating secretion was left intact. (2) In the nanotribometer experiments, a certain contact load (that was in the range of the body weight of the animal and thus simulated the pressure exerted on the tarsi under natural locomotion conditions) was applied to the tarsal pads in a clearly defined manner. Even if the applied liquid film was a bit thicker than that of a non-manipulated pad, the applied pressure in concert with the thinning out of the liquid during friction should have led to the levelling of the fluid film, so that its thickness was certainly comparable to the natural conditions.

### External morphology of adhesive pads

As previously mentioned, the surface of the euplantulae has an imbricate appearance whereby the ledges face distally (Fig. 1D). This could possibly contribute greatly to the observed frictional anisotropy in the push direction on the 3-µm surface. Such cuticular folds acting as ‘friction ridges’ have also been recorded in other cockroaches and might be functionally related to their increased friction in the push direction (Clemente and Federle, 2008; Clemente et al., 2009). However, in our study, these surface structures have only been clearly detected in conventionally critical point-dried samples after the application of a stepwise dehydration procedure in ethanol and an additional cleaning step in hydrogen peroxide. This shows that they are normally masked by the tarsal adhesion-mediating secretion. However, during sliding, the surface film is probably thinned out, so that these structures come into action with the substrate, thus increasing friction. The short trichoid sensilla that are especially present on the euplantulae might function in measuring the normal load and shear stress and might, in this way, be involved in correctly adjusting the tarsal position and the flow of the tarsal adhesion-mediating secretion to current requirements.

### Roughness dependence of cockroach's attachment ability

In these experiments, the effect of surface roughness largely depended on the treatment of the adhesive liquid covering the euplantulae (Fig. 2). The static friction of the non-manipulated tarsi that retain their natural adhesion-mediating secretion remains almost unaffected by the surface roughness, confirming that, in smooth attachment systems, critical roughness effects are not especially relevant in the range of the chosen surface roughnesses and can obviously be compensated by the pliable pad cuticle in concert with the fluid surface film (e.g. Gorb, 2007). However, upon depletion of this fluid film from the tarsal surface by the silica gel plate treatment, friction is considerably decreased on the nanorough (0.05 µm) and the microrough (3 µm) surfaces, whereas it is greatly increased on the rough (11 µm) surface.

On the one hand, these results support the assumption that on nano- and microrough surfaces a major function of the tarsal secretion is the maximization of the effective contact area towards the substrate by filling out its (nano-scale) surface irregularities (e.g. Drechsler and Federle, 2006; Persson, 2007; Gorb, 2007). For effective contact to the 0.05-µm and the 3-µm surfaces, an additional amount of adhesive liquid is obviously necessary. Similar conclusions have been drawn from manipulation experiments conducted on aphids (Dixon et al., 1990) and reduviid heteropterans (Edwards and Tarkanian, 1970). Moreover, technical experiments with rubber on glass have shown that relatively small surface roughnesses are sufficient drastically to reduce adhesion (e.g. Fuller and Tabor, 1975; Persson et al., 2005).

On the other hand, because of the possible dehydration of the cuticle, the silica plate treatment probably made the cuticle of the adhesive pads more brittle, i.e. less compliant (e.g. Klocke and Schmitz, 2011). Apart from the water content of the cuticle itself, Perez Goodwyn et al. (2006) assume that, in orthopterans, the fluid that internally fills the gaps within the cushion-like soft pad cuticle also contributes to its viscoelastic performance. This probably explains the seemingly contradictory increase of the friction force of the treatment with depleted secretion on the rough 11-µm surface (Fig. 2). The reduced pliability of the cuticle might have induced additional micro-wrinkles and allowed surface structures, such as the imbricate surface structure of the euplantulae (Fig. 1D) protrude, more strongly and directly to interdigitate with the protuberances of this especially rough surfaces. Being still deformable, the more protruded (dehydrated) surface structures of the adhesive pads might have led to elongated stretched bands enabling additional elastic energy to be dissipated before fracture occurs (cf. Gay, 2002), possibly even leading to rubber friction (e.g. Persson, 1998). From these considerations it can be deduced that in our experiments the natural tarsal adhesion-mediating secretion has a friction-reducing effect on the rough (11 µm) surface. This can probably be ascribed to the function of the secretion of keeping the adhesive pad cuticle hydrated and thus smooth and pliable. Hence, harsh solid-solid contacts between cuticular surface wrinkles and substrate irregularities are prevented; this might also help to prevent the premature wearing of the adhesive pad material.

In another group of experiments the depleted natural secretion was replaced by squalane or a squalane-based water-in-oil emulsion (Fig. 2). Both these liquids have an oily consistency and are well able to wet the test surfaces. However, according to their exclusively (squalane) or predominantly (biomimetic emulsion) lipoid nature, these treatments are certainly unable completely to restore the previous elasticity of the pad material. Notwithstanding, the application of these fluids obviously prevented the occurrence of rubber friction that we assume to be responsible for the drastic increase in friction in the treatment with depleted secretion on the rough surface. This can be deduced from the reduced friction of the squalane and the squalane-based (biomimetic) emulsion treatment, which, when compared with the non-manipulated tarsi, showed clearly reduced friction forces on the microrough and the rough surfaces (Fig. 2). An alternative explanation for this reduced friction after the application of a thin film of oily liquid might be that these liquids actually cause a lubrication effect, i.e. lower the shear stress. However, the friction of the squalane-coated adhesion pads on the smoothest 0.05-µm surface argues against such an explanation. Instead of a decrease, as expected under a lubrication regime, on this surface, the application of squalane leads to a considerable increase of friction. Assuming that on this almost smooth surface the full compliance of the pad cuticle does not play an appreciable role, the observed increased friction forces should be mainly determined by the bulk properties of the applied fluid. Similarly to squalane, the application of the biomimetic squalane-based ‘SG4’ emulsion leads to a significant increase in static friction on this nanorough surface compared with both of the rougher surfaces. Notwithstanding, on the nanorough surface, its friction is much lower than that of pure squalane (Fig. 2). An increased friction of squalane with respect to the squalane-based ‘SG4’ emulsion has also been confirmed by Speidel et al. (2017) in their tribological analyses of the rheological bulk properties of bioinspired synthetic ‘insect adhesives’. Whereas squalane is an ideal Newtonian fluid without a yield point, the squalane-based ‘SG4’ emulsion behaves in a non-Newtonian way and, in agreement with its yield stress, is a Bingham fluid (e.g. Derkach, 2009; Douaire et al., 2014). The admixture of the gelatin-containing water phase makes up 50% of the total volume of the emulsion. According to emulsion theory, such relative amounts of the dispersed phase determines the overall tribology of the system and usually results in an increase of the viscosity of the emulsion (e.g. Lee et al., 1997; Kutz et al., 2011). However, on studying oil continuous emulsions in a soft steel-elastomer contact tribology regime, Douaire et al. (2014) show that increased phase volumes lead to increased friction only in the elastohydrodynamic regime, whereas in mixed and boundary regimes, lubrication is improved. According to these authors, emulsions support the load better than the pure oil because of their higher viscosities and thus prevent excessive friction between the tribopairs. For all these reasons, we conclude that our experiments actually occurred in the boundary or mixed friction regime being characterised by low to moderate entrainment speeds and viscosities and usually thin fluid films (Douaire et al., 2014). This corresponds well to the conditions that we expect for insect adhesive systems and explains the increased friction of purely squalane-covered compared with the emulsion-covered feet, and also the natural secretion achieved on the nanorough test surface.

The previous considerations suggest a twofold function of the emulsion nature of insect tarsal adhesion-mediating secretions during locomotion. On nanorough surfaces, insects appear to benefit from employing emulsions instead of pure oils to avoid excessive friction forces (see comparison between squalane and biomimetic emulsion in Fig. 2). From this it can be concluded that if thin films of pure liquid oils were used by insects in their tarsal secretions, these would not only impede effortless detachment of the tarsi, but also advance wear and tear. On rougher surfaces, friction is usually reduced per se, so that, in this case, the main function of the tarsal adhesion-mediating secretion might be to improve surface contact by keeping the cuticle compliable (also by retarding excess evaporative water loss via a protective lipoid shield) and (if sufficiently fluid) compensating surface irregularities of the substrate (e.g. Drechsler and Federle, 2006; Persson, 2007).

### Friction depending on the absence and presence of tarsal adhesion-mediating fluids

Our nanotribometric friction measurements of the second euplantula were performed on the microrough (3 µm) surface only. The applied normal load of 5 mN was well within the range of the load that is experienced by a single tarsus when the 5-10 g heavy animal is standing on a planar surface. The results largely correspond to those obtained in the centrifugal force experiments as discussed in the previous section. Whereas the non-manipulated euplantulae showed the highest static and sliding friction values of all the treatments, friction was significantly reduced in the euplantulae when their adhesion-mediating secretion had been depleted by the silica gel plate treatment (such a reduction could not be confirmed only in the middle tarsus; since the time interval between the friction measurement of the fore and the middle tarsus amounted to 1-3 h, in this case, there might have been sufficient time to allow the recovery of the initial secretion volume) (Fig. 4). In correspondence with our friction measurements measured with an ‘insect centrifuge’, the replacement of the natural tarsal secretion by squalane and the bioinspired squalane-based emulsion led to a reduction of the friction force to a similar extent as in the treatments in which the tarsal secretion had been depleted from the euplantulae. According to our previous discussion, this reduction may be attributed to an impaired compliance of the pad cuticle after partial dehydration, which cannot be fully regained by the application of lipoid liquids. This frictional impairment by the treatments was more pronounced in the push than in the pull direction (although, in several cases, the pull direction was also effected, albeit to a lesser extent), so that in six out of nine cases, the treatments led to a diminishment of the observed frictional anisotropy between both the sliding directions (Fig. 4). In the cockroach *Nauphoeta cinerea* on smooth surfaces, Clemente and Federle (2008) have ascribed the direction-dependent friction forces of the adhesive pads to changes in their real contact area; these changes are considered to result from the specific arrangements of the non-fixed (‘footloose’) tarsal chain during pushing or pulling. In experiments on the fixed tarsi of the same species, Clemente et al. (2009) have established higher friction forces in the push direction but only on 4-µm surfaces and not on smoother ones. In this case, the authors have attributed this directionality to the interlocking of the surface irregularities with the ‘friction ridges’ established on the euplantula surface. In the same cockroach species, Zhou et al. (2015) have found that pulling shear forces directed towards the body improve the compliance of the arolium and, thus, improve their capabilities on surface microstructures. On consideration of our results attained on fixed tarsi, we conclude that, in *G. portentosa* on microrough surfaces, the cuticular pad material itself together with its tarsal adhesion-mediating secretion is able to generate higher friction forces in the push direction (Fig. 4: ‘natural secretion’). Possible causes for this directionality can be deduced from the friction of our three types of surface manipulations with respect to the tarsal secretion, although their outcomes are not always consistent among the fore, middle and hind tarsus. In the fore tarsus, all three types of treatment result in the disappearance of the anisotropic friction behaviour. This result indicates the significance of the compliance of the pad cuticle, which is important for maximizing the real contact area and thus the adhesion-induced friction, namely in the push direction. Hence, with respect to the findings of Zhou et al. (2015), the proximal euplantulae of cockroaches might show the opposite behaviour to that of the arolium, enhancing their compliance during distally directed shear stresses. In the hind tarsus, almost the same pattern has been observed as in the fore tarsus (all friction values reduced compared with the non-manipulated tarsus).

Using a relatively low normal load of 0.6 mN, Speidel et al. (2017) have determined the friction of both the simulated adhesives (squalane and the squalane-based biomimetic w/o emulsion ‘SG4’) in a nanotribometric setup between two hard surfaces. These liquids showed shear stresses between 41 Pa (‘SG4’emulsion) and 155 Pa (squalane), far below the shear stresses attained in our experiments (3.7 kPa) in which these fluids were directly applied to the euplantulae. First, these differences can be assigned to the much thinner film thickness that comes into action in naturally walking insect tarsi and that enhances friction by several possible mechanisms (see discussion in Federle et al., 2004). Second, both the soft pad cuticle and the adhesion-mediating secretion provide a soft tribology system, whose friction is enhanced by the energy dissipating properties of the (visco-) elastic pliable cuticle (see discussion in Gorb, 2007).

### Adhesion of euplantula depending on the absence and presence of tarsal adhesion-mediating fluids

Adhesion was measured at the same surface roughness (3 µm) and almost the same normal load as friction. Our results show that, in non-manipulated euplantulae, adhesion is kept in a moderate range showing adhesive tenacities between 25-58 Pa, whereas the applications of squalane and the squalane-based emulsion result in two- to tenfold higher adhesion forces. Compared with friction, the adhesion forces are lower by one to two orders of magnitude. In non-manipulated tarsi that had retained their natural adhesion-mediating secretion, friction exceeded adhesion by two orders of magnitude. This supports the view that, at least on microrough surfaces, the proximal euplantulae of *G. portentosa* are functional mainly for effective friction rather than for adhesion acting perpendicularly to the substrate. This function can also be deduced from the distally directed hexagonal surface pattern of the euplantulae (Fig. 1D) that probably functions to enhance friction in the push direction. In smooth attachment systems, reduced perpendicular adhesion in comparison to tangential friction has also been found in the arolia of weaver ants (Federle et al., 2002, 2004) and the euplantulae of stick insects (Labonte and Federle, 2013). This can be probably mainly assigned to the strong elastic anisotropy (cf. Scholz et al., 2008 for the arolium of stick insects) of the pad cuticle that probably shows a limited dorso-ventral expansibility (i.e. in the normal pull-off direction) leading to higher tensile rigidities in this direction compared with the proximal-distal direction (i.e. the parallel friction direction). The limited expansibility in the dorso-ventral direction is probably determined by the vertical orientation of the cuticular rods that have been described in many studies of the ultrastructure of smooth adhesive attachment pads (e.g. Gorb et al., 2000; Perez Goodwyn et al., 2006) including the tarsal pads of *G. portentosa* (C. S. and O. B., unpublished data). With respect to our various treatments, the depletion of the tarsal secretion diminishes tarsal adhesion by up to 25% in comparison with the non-manipulated tarsi (Fig. 3), demonstrating that the adhesion-mediating secretion considerably contributes to the adhesion of the euplantulae on microrough surfaces. On the other hand, this result shows that the compliant pad cuticle has its own tackiness, which can be ascribed to the viscoleastic properties of at least its internal layers (cf. Gorb et al., 2000; Scholz et al., 2008; Bennemann et al., 2014). The physical mechanisms that generate adhesive forces during perpendicular pulls are usually attributed to capillarity and viscosity (cf. Federle et al., 2002; Bhushan, 2003; Betz and Kölsch, 2004; Persson, 2007). Borrowing models from fracture mechanics and based on the recent experimental data on the arolia of stick insects, Labonte and Federle (2015) suggest that neither of these mechanisms play a decisive role in insect adhesion. Rather, the secretion injected into the space between the tarsus and substrate is considered to function in minimizing viscous dissipation and thus to facilitate easy detachment during locomotion. This view is supported by recent chemical analyses of the tarsal adhesion-mediating secretion of the locust *Schistocerca gregaria* and the cockroach *G. portentosa* suggesting a semi-solid (grease-like) consistency of the adhesive instead of a liquid one (Reitz et al., 2015; Gerhardt et al., 2015, 2016). This is further supported by our adhesion experiments (Fig. 3). Whereas our data show a slight decrease in adhesive tenacity in euplantulae with depleted secretion, adhesion is considerably increased after the application of squalane or the squalane-based water-in-oil emulsion. Although a long-chained (C30) hydrocarbon, squalane has an oily consistency at room temperature, which is attributable to its several methyl branches. Its viscosity amounts to 30 mPa s. This means that, in our adhesion measurements, according to their fluid consistence, both these liquids were well able to wet the substrate for good adhesion. Moreover, it can be assumed that the capillary greasy film (showing high segmental mobility) is dragged out between the two surfaces upon their separation, thus largely dissipating the separation energy and increasing the adhesion. This is in correspondence with the probe tack test of Speidel et al. (2017); this test has revealed considerable adhesive tenacities in the squalane-based ‘SG4’ emulsion. The influence of viscous dissipation in these experiments can be deduced from the finding that this emulsion shows significantly higher adhesion forces than pure squalane. Identical results have been found in the soft tribology regime of our present contribution (Fig. 3).

**Fig. 6.**
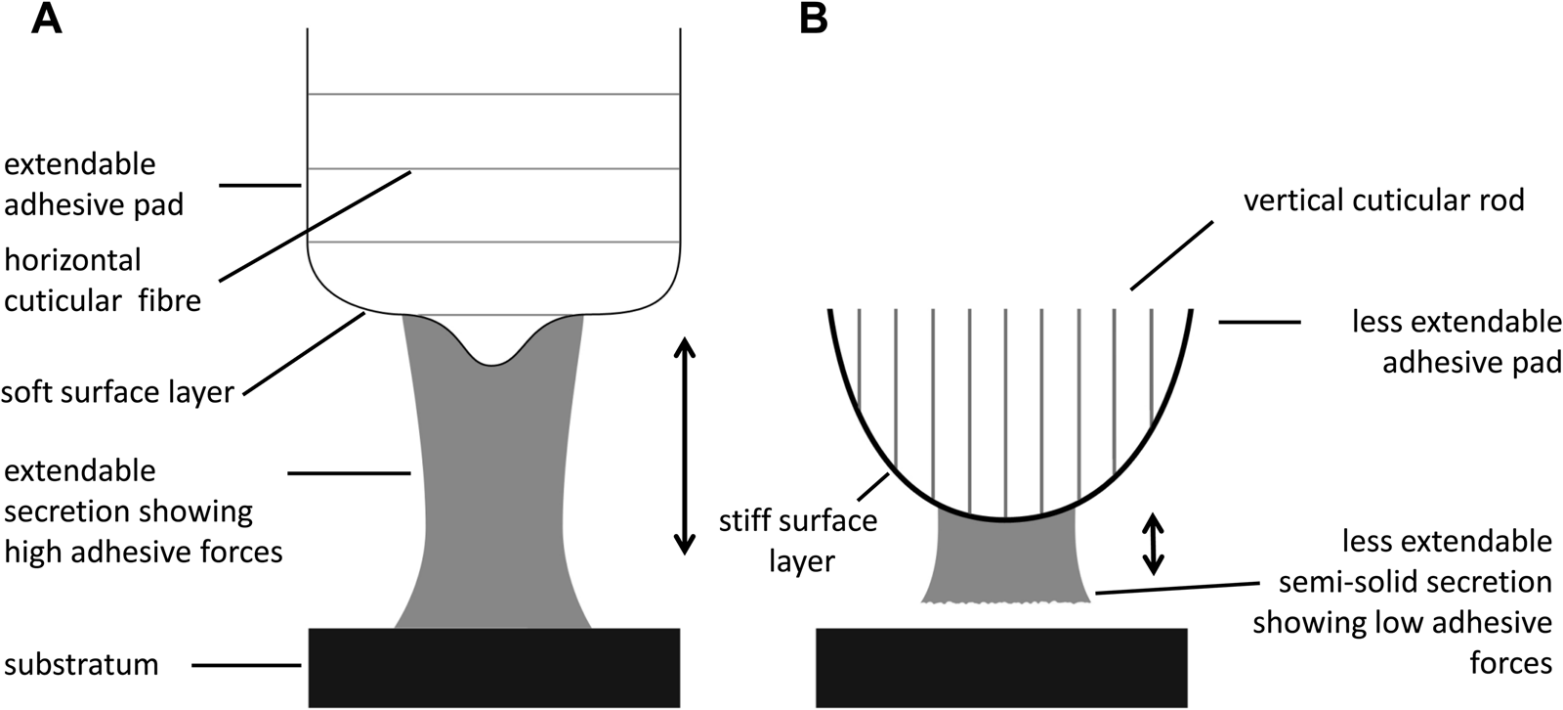
**Schematic comparison between two principally different systems of energy dissipation in insect adhesive organs during a pull-off situation (pull-off symbolized by double arrow).** (A) Viscous dissipation enhancing system (e.g. adhesive paraglossae of *Stenus* beetles). A viscous liquid or gel-like adhesive that shows good adhesion properties is extended upon separation of both the surfaces without premature rupture, thus provoking viscous dissipation. The drag of the fluid is transferred to the pad cuticle whose upper layer is considered to be very soft and compliable, deforming viscoelastically in the direction of the pull. The elongation of the entire pad upon pull-off is further supported by the horizontal arrangement of the cuticular fibres within the pad. (B) Adhesion and dissipation reducing system (e.g. euplantula of cockroaches). The separation of both the surfaces is accompanied by the rapid adhesive failure of an adhesive surface film, which is caused by its low wetting properties due to its semi-solid consistence. This behaviour is supported by an increased tensile strength in the pull direction caused by an enhanced stiffness of the outer layer of the pad cuticle together with a vertical orientation of the internal cuticular fibres or rods.

The natural tarsal adhesion-mediating secretion behaves quite differently from the oily squalane. According to its semi-solid consistence, its wettability towards the substrate should be largely reduced, impeding adhesion. This shows that our ‘biomimetic’ squalane-based ‘SG4’ emulsion is actually not capable of mimicking one major property of the natural adhesive, i.e. the combination of low adhesive forces with moderate friction. Such combination might be better attainable by enriching a waxy bulk with certain plasticisers. The presumed adhesion-reducing effect of the adhesive is functionally supported by the structural peculiarities of the cuticle itself, namely the previously mentioned perpendicular arrangement of the cuticular rods and fibres within the adhesive pads (enhancing tensile strength in the vertical direction) and the consistency of the outer tarsal cuticular layer, which is supposed to be stiffer than previously assumed, surpassing the elastic moduli of the inner cuticular layers (Bennemann et al., 2014). All these conditions might impair the transfer of the viscous dissipation of the adhesive to the viscoelastic pad cuticle. In Fig. 6, we schematically compare two principally different systems of energy dissipation in insect adhesive organs during pull-off. Fig. 6A shows a case in which good adhesion to the substrate in combination with viscous (tarsal adhesion-mediating secretion) and viscoelastic (pad cuticle) dissipation are used to maximize adhesive strength. This case is found in the labial sticky paraglossae of staphylinid beetles of the genus *Stenus* during prey-capture (Kölsch and Betz, 1998; Koerner et al., 2012a,b: Fig. 6). Fig. 6B represents the situation assumed in the tarsal euplantula of *G. portentosa* during the release phase in normal walking. In this case, adhesion is largely reduced, facilitating easy detachment from the ground. However, other insects might overcome high tarsal adhesion forces during locomotion by employing particular kinematics and/or exerting ‘highly energetic’ detachment movements.

Speidel et al. (2017) have shown that low viscous dissipation of the bulk adhesive can be readily attained by appropriately prepared oil-in-water (o/w) emulsions. However, for insect tarsal attachment pads, o/w emulsions would be in conflict with the demand to withstand water loss; indeed, Federle et al. (2002) and Dirks et al. (2010) have presented evidence that, in insect tarsal secretions, a watery phase is dispersed within a continuous lipid phase. In insects, one structural principle that reconciles all these functions is the synthesis of waxy semi-solid (grease-like) water-in-oil secretions. In addition to slip resistance, easy tarsal release and desiccation resistance, such adhesives might also help to protect the adhesive pad from abrasive damage.

### Frictional directionality of euplantula and arolium

The direction dependency of adhesive structures is considered a general property of insect tarsi (e.g. Bullock et al., 2008; Clemente and Federle, 2008). In this final experiment, we compared the friction coefficients of the euplantula and the arolium with respect to possible directionality effects (Fig. 5). In the euplantula of both the fore and the hind tarsus, we found about twofold (hind tarsus) to sixfold (fore tarsus) higher friction values in the push direction. This is in accordance with the locomotory function of these legs. Whereas the fore leg is mainly adapted to adsorb energy when stepping out (in correspondence to the force vector of the fore leg), the middle and hind legs provide the major thrust, pushing the animal forward (Full et al., 1991, 1993, 1998). The friction anisotropy of the euplantulae of the fore leg in the push direction would not bring about a supporting effect only during upward locomotion (upward vertical climbing). Instead, in this situation, according to their pull directionality, the arolia of the fore legs can exert an additional drag force, i.e. they behave in an opposite manner to the euplantulae (Fig. 5). In the hind leg, the arolia do not show such directionality. Here, the arolia exhibit equal high friction forces in the push and pull direction.

In the cockroach *Nauphoeta cinerea*, Clemente and Federle (2008) have found that the front legs mainly employ the arolium when climbing upwards, but the euplantulae when walking downwards. From their experiments, the authors conclude that the direction-dependent forces result from changes in contact area rather than pad efficiency. In addition, at least in our experiments performed on a microrough surface, the anisotropic imbricate surface structure of the euplantulae is probably involved in the generation of a higher friction force in the push direction (Fig. 1D). Such effects of distally directed ‘friction ridges’ have also been discussed for the euplantulae of *N. cinerea* (Clemente et al., 2009). Moreover, several authors (e.g. Gorb and Scherge, 2000; Gorb, 2007; Zhou et al., 2014) hint at the importance of the fibrous inner structure of smooth attachment pads for generating directional friction forces. The euplantulae of *N. cinerea* are internally made up of numerous branched rods oriented distally at an angle to the surface (cf. Fig. 1d in Clemente and Federle, 2008). This is opposite to the orientation of such rods in the bushcricket *Tettigonia viridissima*; its rods are sloped in the proximal direction (cf. Fig. 13 in Gorb, 2007 citing Gorb and Scherge, 2000). Since Gorb and Scherge (2000) have measured increased friction in the pull direction, this structural difference might account for the opposite directionalities found in cockroaches versus orthopterans.

In terms of the arolia, our SEM studies did not reveal any anisotropic surface topography that might account for the observed back and forward directionality of the tarsi. For the increased shear forces established in the arolia of *N. cinerea*, Zhou et al. (2015) suggest some form of change in the cuticular pad material possibly related to the internal fibre structure. It is possible that in *G. portentosa* the observed anisotropy of the arolium is attributable to its unfolding in the preferential direction causing an enlargement of its contact area. Overall, our results correspond well with previous analyses of the cockroach *N. cinerea* (Clemente and Federle, 2008; Clemente et al., 2009) and suggest that the euplantulae of *G. portentosa* are more specialized for pushing, whereas the arolia of the fore tarsus perform better in pulling.

### Conclusions

The present study demonstrates that the smooth attachment system of the cockroach *G. portentosa* combines a bundle of properties that make it possible to cope with quite different demands arising during locomotion. A division of labour between the euplantulae and arolium with respect to the frictional directionality in the push versus pull direction has previously been emphasised for other insects, including cockroaches (e.g. Labonte and Federle, 2013). Whereas the euplantulae appear to increase friction (namely, in cockroaches, mainly in the push direction), other studies (e.g. Clemente and Federle, 2008) have revealed that the arolia, in addition to increasing friction mainly in the pull direction, are important in (upside-down) situations in which adhesion forces are required. This is in contrast to the euplantulae, which need to diminish adhesion to be able easily to detach their tarsi from the substrate during normal walking. Our study shows that not only morphological peculiarities of the pad cuticle are responsible for this behaviour, but also the emulsion-like secretion that covers the euplantulae. According to its semi-solid consistency, its adhesion to the substrate during vertical pull-off is diminished (cf. Fig. 6B). During friction, according to its emulsion nature, the thin secretion film seems to adopt the function of a lubricant, thereby preventing immoderate friction forces. This seems to be especially important on smooth to nanorough surfaces. On rougher surfaces, other functions of the secretion such as the amplification of the true contact surface by keeping the cuticle compliable become more important. This demonstrates that the specific functional properties of the adhesive tarsal secretion in insects need to be considered context dependently, requiring the combination of various experimental and methodological approaches.

The development of bioinspired adhesive systems might greatly benefit from such investigations (cf. Speidel et al., 2017). If combined with microstructured adherents (e.g. technical polymer foams as porous carrier materials), emulsion-based adhesives should make possible the fine-tuning of bonding technological properties, especially in the range of low adhesive forces. Possible fields of application are initially non-sticky tapes whose adhesiveness can be activated by compressive stress, fluid-supplied medical patches and adhesively controllable capillarity-based adhesion devices (e.g. Vogel and Steen, 2010).

## MATERIALS AND METHODS

### Test substrates: surface roughness and wettability measurements and treatments/modification

**Fig. 7.**
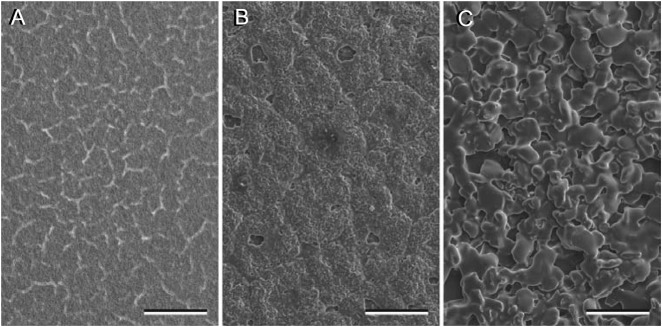
**Scanning electron micrographs of silanized aluminum oxide grinding discs of three different roughnesses (R_a_) as used in the experiments.** (A) 0.05 µm, (B) 3 µm, (C) 11 µm. The surfaces were photographed at identical magnifications. Scale bars: 100 µm.

The aluminum oxide (Al_2_O_3_) grinding discs (FibrMet Abrasive Discs, Backing: PSA, Buehler, Lake Bluff, IL, USA) used in our experiments were selected for three nominal asperity sizes, i.e. 0.05 µm, 1 µm, and 30 µm (asperity sizes according to manufacturer information) (Fig. 7). The surface roughness parameters *R_a_*, *R_z_* and *R_t_* (cf. Gadelmawla et al., 2002) were determined using a white light sensor of the optical profiler MicroProf (FRT, Bergisch Gladbach, Nordrhein-Westfalen, Germany) and the software Aquire (FRT, Version 1.82) for the measurement and FRT Mark III (FRT, Version 3.9 R3T1) for analyzing the ROI data. The size of the scan window amounted to 0.25 mm², the resolution was 10 nm (vertical) and 1-2 µm (lateral), respectively. The surface parameters were calculated from the ROI data according to the DIN EN ISO 11562:1998 standards. Five surfaces per particle size (*n*=5) were tested using measurements at three randomly chosen locations per surface.

In order to provide otherwise comparable surface chemistries, the discs were treated by silanization involving (3-aminopropyl) triethoxysilane to create relatively hydrophobic surfaces. The activated (3-aminopropyl) triethoxysilane with a silan proportion of 20% was made up into a solution consisting of 5 ml reagent, 10 ml double distilled water and ethanol (pH 4.9). After being stirred for 5 min, the solution was poured over the grinding discs that were stored separately in Petri dishes. The discs were rotated for 48 h at 40°C in a rotary evaporator. Finally, the modified discs were washed with isopropanol and cured in a cabinet drier at 80°C for 30 min. Hydrophilicity and surface free energy of the silanized discs were accessed by contact angle measurements of 2 µl drops of Millipore-filtered water, diiodomethane and ethylene glycol by means of a drop shape analysis system (DSA 10Mk2; Krüss, Hamburg, Germany). The contact angles were calculated from the respective entire 15 drop shapes of each liquid by the circle-fitting method (DSA software version 1.91.0.2, Krüss, Hamburg, Germany). The surface-free energies were calculated applying the Owens-Wendt method (Owens and Wendt, 1969).

### Scanning electron microscopy

For scanning electron microscopy (SEM), entire animals were fixed in 70°C hot ethanol (70%) for 10 min, stepwise dehydrated in ethanol (up to 100%), critical-point dried (Polaron 3100, Quorum Technologies, Laughton, East Sussex, UK), fixed onto stubs, coated with gold (Emitech K550X, Quorum Technologies) and investigated with a Zeiss EVO LS10 scanning electron microscope (Carl Zeiss, Oberkochen, Baden-Württemberg, Germany). In order to improve the visibility of possible surface pattern of the adhesive pads, a set of tarsi were critical-point dried and then cleaned in hydrogen peroxide (cf. Bolte, 1996) and briefly immersed in 100% ethanol; superfluous liquid was removed by filter paper once the sample had been withdrawn from the ethanol.

### Centrifugal force experiments on differently rough surfaces

A hand-manufactured ‘insect centrifuge’ modified after Bohn et al. (2011) was used to determine the attachment of the entire insect with all tarsi attached to the surface. For the experiments, the silanized grinding discs were attached to the centrifuges' rotary disc (diameter 21 cm) with double-sided adhesive tape. As soon as the centrifuge started to accelerate, physical forces acted on the cockroaches on top of the discs. The centrifugal force F_cf_ pushed the animal outwards and worked against the opposite centripetal force F_cp_ that was directed towards the rotation centre. The moment before the cockroach started to slip off the disc, it experienced a centripetal force F_cp_, which was equated with the maximum static friction force F_s (max)_. At this point, the image of the video was selected to analyse the speed and the distance between the mesothorax (i.e. the estimated centre of mass) of the cockroach to the rotation centre.

Friction was measured under four different treatments: (1) leaving the tarsal adhesion-mediating secretion intact, (2) after depletion of the adhesion-mediating secretion by letting the animals run for 30 min on silica gel plates [reversed-phase (RP) modified silica TLC plates, RP-8 F254D, Merck, Darmstadt, Germany] that had been dried at 60°C for 30 min prior to the measurements, (3) after replacement of the natural adhesive by the hydrocarbon squalane (C_30_H_62_) (Sigma-Aldrich, Munich, Bayern, Germany) and (4) after replacement of the natural adhesive by the bioinspired synthetic w/o emulsion prepared from squalane and gelatin (25.6 g squalane, 10.8 g Span 80) detergent (sorbitan monooleate, for synthesis, Carl Roth), 0.11 g sodium di(ethylhexyl)sulfosuccinate (AOT) (Sigma-Aldrich), 14.6 g water containing 4 mg sodium azide as a biocide (NaN_3_, Sigma-Aldrich), 1.0 g gelatin (Sigma-Aldrich) (cf. Speidel et al., 2017: emulsion ‘SG4’). For the centrifuge experiments, treatments (3) and (4) were applied to the euplantulae after the depletion of the natural adhesion-related secretion (2) as follows. A Petri dish was covered with a filter paper (Quality Filter Paper 413, diameter 9 cm, VWR, Radnor, PA, USA) and 500 µl (squalane) or 700 µl (synthetic ‘SG4’ emulsion) of the artificial adhesive was pipetted on it and evenly distributed with a razor blade. A cockroach was clamped between thumb and forefinger and pushed onto the filter paper, so that only the euplantulae and the arolium of the tarsi touched its surface. Subsequently, the cockroach was placed in the centre of the disc in the way that the head showed towards the centre. The centrifuge was immediately set in motion to prevent the animals from walking around and scattering the artificial secretion. The foot prints of the tarsi left on the aluminium oxide discs confirmed the successful application of the adhesive. After three individuals had been tested, the discs were replaced. The recovery times of each individual amounted to two days between treatments (1) and (2), 24 days between treatments (2) and (3), and 10 days between treatments (3) and (4). Cryo-SEM controls confirmed the depletion of any secretion on the attachment pads due to the silica gel treatment and did not reveal any contamination of their surfaces by the silica. Moreover, these controls did not reveal any differences between the non-manipulated and the emulsion-treated euplantulae; in both cases, the surface structures of the adhesive pads were masked by the adhesion-mediating secretion.

In total, 10 adult males (6.43±1.2 g) and 10 adult females (8.10±1.5 g) (arithmetic means±s.d.) of *G. portentosa* (*n*=20) of about the same age and with no visible (age-related) damage of the adhesive pads (cf. Zhou et al., 2015) were taken from a laboratory colony and kept in individual plastic cages (20 cm×10 cm×6 cm) with moistened plaster of Paris (mixed with activated charcoal to prevent mould formation) as the ground material and a paper towel (Zewa, Mannheim, Baden-Württemberg, Germany) covering the plaster of Paris. Under anaesthetization with CO_2_, the cuticular claws of the cockroach were removed with a scalpel to prevent their interference with the attachment of the euplantulae and arolia. Just prior to a measurement, each test individual was weighed.

Five test runs per individual, treatment and surface were recorded. Out of the five test runs, the three most precise videos were chosen to calculate an arithmetic mean of force values for each individual. The recorded centrifuge experiments were screened with the software Adobe Premiere Pro (Adobe Systems 2003) to determine the maximum friction force (*F_s_^max^*=*F_cp_*) as indicated by the frames in which the cockroaches slipped off the rotation disc. The frames were edited via the software ImageJ and the distance between the mesothorax of the cockroaches and the centre of the rotation of the disc was measured. Together with the velocimeter speed displayed in the selected frame and the body mass, these data were used to calculate the dimensionless safety factor as
(1)
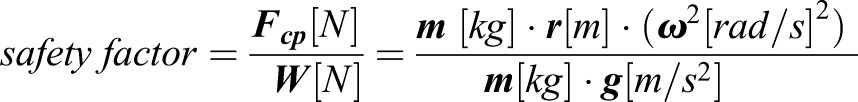
where ***F_cp_***=centripetal force, ***W***=body weight, ***m***=body mass, ***g***=acceleration of gravity, ***r***=distance between the mesothorax of the cockroach and the centre of rotation of the disc and ***ω***=angular velocity.

### Nanotribological measurements on single euplantulae

For this experiment, both the maintenance of the animals and the application of the four treatments were the same as that described above. However, the claws were left intact, since they did not get in contact with the test surface. In this experiment, only the test surface showing a roughness of 3 µm was used. Treatments (3) and (4) (see previous experiment) were applied to the euplantulae of the mounted specimens after the depletion of the natural adhesive (treatment 2); 100 µl of the artificial adhesives was deposited on the euplantulae with a hair pencil prior to the experiment.

For the measurements, adult *G. portentosa* cockroaches were anaesthetised with ethyl acetate and CO_2_ and positioned in a self-fabricated mount made of duroplast. The animals were fixed by means of a combination of Parafilm (Bemis Company, WI, USA), insect pins and adhesive tape. The tarsi were glued (UHU supergel, Bühl, Baden-Württemberg, Germany) with their dorsal side onto a microscope slide, so that their adhesive pads (euplantulae, arolium) were exposed away from the animal. Tribological measurements were performed with the nanotribometer NTR^2^ (CSM Instruments, Peseux, Neuenburg, Switzerland) equipped with the dual beam cantilever STH-001. This cantilever featured a highly sensitive dual beam spring able to measure forces in the x (F_t_ Stiffness 4.8139 mN/µm) and z (F_n_ Stiffness 0.5122 mN/µm) directions with a resolution of 30 nN. Both adhesion and friction forces were detected by two independent high-resolution capacitive sensors, whereas a piezo actuator provided smooth and steady motion at a slow pace. The measuring heads consisted of cylindrical aluminium pins to which, on their terminal ends, the silane treated Al_2_O_3_ polishing paper plate was glued. This test surface showed a surface roughness of 3 µm (cf. Table 1) and a slightly non-polar surface energy of 33.3 mN m^−1^ (cf. Table 2). For adhesion, the polishing paper plate formed a circular contact surface 25.1 mm², whereas for friction a rectangular contact surface of 1 mm² was used. In any case, the surface area of the measured adhesive pads was lower than the applied test surface, so that the occurrence of possible edge effects caused by a too small tester can be excluded. All experiments were carried out at room temperature (ca. 22°C) and a relative humidity of ca. 50%. For the euplantulae, adhesion and friction were determined for five males and five females (*n*=10) on all three tarsi (fore, middle and hind tarsus).

We assume that the convex surfaces of both the arolium and the euplantula were levelled out upon the applied contact pressure and did not affect our results.

#### Adhesion measurements

Adhesion was simultaneously determined on all four euplantulae by pressing the measuring head onto the ventral side of the tarsus followed by continuously pulling it away perpendicularly to the surface. Once the silanized polishing paper surface touched the euplantulum with a contact load of 1 mN, the measurement started. The pressure was then increased up to 8 mN with a loading rate of 0.3 mN s^−1^. After another 10 s, the pressure decreased with an unloading rate of 0.3 mN s^−1^. At a load of 0.3 mN, the circular Al_2_O_3_ plate was retracted with a speed of 20.0 μm s^−1^. The lowest value representing the pull-off or adhesion force was estimated after setting the baseline at an arbitrary value before any pressure was applied (Microsoft Excel, Microsoft Office 2010). For further statistical analysis, we used the means of three succeeding measurements per specimen. To refer the measured adhesion force to the apparent surface area of the euplantulae, the tarsi were fixed as described above, so that their euplantulae were freely exposed. We then applied cover glasses to the euplantulae and loaded them with 2 g, 5 g, 10 g, and 30 g weights. Using a binocular microscope (Leica MZ 12.5, Leica Microsystems, Wetzlar, Baden-Württemberg, Germany) combined with a digital camera (AxioCam, Mrc5, Carl Zeiss Microscopy, Jena, Thüringen, Germany), we took images of the euplantulae and determined their contact surface area by means of the software AxioVison (Carl Zeiss Microscopy). We applied these measurements separately to the fore, middle and hind leg of three female and three male individuals and drew a calibration curve to determine the mean contact area of each sex for the applied load of 8 mN.

#### Friction measurements

The friction forces were determined for the second (not distinctly lobed) euplantula. The rectangular Al_2_O_3_ plate was dragged in parallel over the surface with a load of 5 mN and a velocity 10 µm s^−1^. For a measurement, the measuring head moved with an oscillated motion of the measuring table of five cycles over the surface, whereby one cycle equated to one forward (distad: *push*) and backward (proximad: *pull*) motion by a distance of 100 µm in each direction. In Microsoft Excel, the coefficient of static friction was extracted as the maximum absolute value from the data, whereas the coefficients of sliding friction were extracted as the means of the sliding distance between the maximum absolute values and the change in direction of a cycle. For further statistical analyses, the arithmetic means of the coefficients of static and sliding friction of the three selected middle cycles of a measurement were taken.

### Comparative direction-dependent nanotribological measurements with single euplantulae and arolia of fore and hind tarsi

This experiment was conducted to compare the friction of the euplantulae and the arolium with respect to possible anisotropies in the push and pull direction. The circular Al_2_O_3_ plate was dragged in parallel over the surface of the first (not distinctly lobed) euplantula and the arolium, respectively, with a load of 5 mN and a velocity 25 µm s^−1^. The test surface area amounted to 3.2 mm². Otherwise, the conditions were identical to those of the previous experiment. In the case of the arolium of the hind tarsus, seven males and seven females (*n*=14) were tested, in all other cases five males and five females (*n*=10).

### Statistical analysis

For statistical analysis, all data were ln-transformed. If values between 0 and 1 occurred in the data set, the value 1 was added to each value prior to transformation. We used the same individuals for measuring the effects of the various treatments, so that we could use test statistics for repeated measurements by comparing the different treatments. For evaluating the general influence of the main factors including their interactions, we applied linear mixed models for repeated measures to our ln-transformed data. After these general tests, pairwise *a posteriori* tests were performed. We used paired *t*-tests, if the data had previously been visually confirmed as having a normal distribution. Alternatively, if several frequency distributions clearly differed visually from a normal distribution, we performed pairwise Wilcoxon tests. For all the statistical analyses, we used the software IBM SPSS 23 (SPSS, Chicago, IL, USA). Our *a posteriori* tests referred to a limited number of pairwise comparisons of single treatments or factors keeping all other parameters constant. Since the outcomes of these tests were not used to reject or retain a superordinate global hypothesis, a correction for multiple testing was not required.
